# Exploring evolution of brain genes involved in microcephaly through phylogeny and synteny analysis

**DOI:** 10.1186/1742-4682-10-61

**Published:** 2013-10-22

**Authors:** Sobiah Rauf, Asif Mir

**Affiliations:** 1 Department of Bioinformatics & Biotechnology, International Islamic University, Islamabad, Pakistan

**Keywords:** MCPH, Phylogenetic analysis, Evolutionary relationship, Synteny, Conservation

## Abstract

**Background:**

Human brain development is a complicated process. When normal growth and development of brain or central nervous system is impaired, it leads to neurodevelopemental disorders (NDDs). Autosomal Recessive Primary Microcephaly (MCPH) is one of those, for which seven loci (MCPH1-MCPH7) with the corresponding genes (MCPH1, WDR62, CDK5RAP2, CEP152, ASPM, CENPJ, and STIL) have been reported so far. An important field of study is to find out diversity among organisms due to evolution. How species are related to each other can be inferred through finding evolutionary relationship between organisms in the form of ancestors and descendents.

**Methods:**

MEGA5 was used for phylogenetic tree reconstruction. Pair-wise and multiple alignment was built through ClustalW algorithm. Neighbor joining (NJ) and maximum parsimony (MP) methods were used for tree reconstruction. Bootstrap analysis was done to check the reliability of trees. Synteny analysis was performed using Ensemble synteny view in ensemble database and genome synteny viewer (GSV).

**Results:**

Evolutionary time for single gene trees showed that CENPJ (0.02) evolving rapidly while CDK5RAP2 (0.1) evolving with least rate as compare to other genes. All trees were reconciling the species divergence time. Chimpanzee was inferred as closest specie of Human. In MCPH combined tree, five duplications were observed. Four duplications were before and one was after vertebrate and invertebrate divergence. Two genes MCPH1 and WDR62 were closely related with each other. Synteny analysis indicated that maximum conservation of Human was with Chimpanzee. Highly conserved synteny was observed for Human and Chimpanzee in case of CENPJ with no deletion.

**Conclusion:**

It has been hypothesized that due to having closest relationship, mutations can affect Chimpanzee likewise as these affect Human. Conservation shows that apart from sequence similarity, function of MCPH genes in closely related species is also same and this function disrupts as a result of mutation and hence leads to the diseased state. Huge genomic and proteomic data is available today which enables us to perform *In Silico* analysis. Our cost and time effective analysis has opened many insights into disease understanding and it will definitely provide a way towards accurate diagnosis.

## Background

Evolution is the change that leads towards the diversity. This diversity can be at any biological level including species, organisms, and also at molecular level i.e. DNA and Proteins [1]. An important evolutionary study is the reconstruction of phylogenetic trees. Phylogenetic tree reconstruction is to estimate the evolutionary relationship between organisms. From genetic sequence data, trees can be reconstructed using many different techniques. The relationship is represented in the form of a branching tree sort of diagrams showing ancestors and the evolved descendents [2].

There are two strategies for reconstruction of these trees [3]:

Exhaustive-search which examine all possible trees or their large number and finally select the best one on the basis of certain criterion or threshold for example Maximum-parsimony (MP) method [4], the Fitch-Margoliash (FM) method [5], the maximum-likelihood (ML) method [6] and Bayesian approach [7]

Stepwise clustering method which constructs the best tree in a step wise fashion after examining local topological relationships of a tree. Example of this category is neighbor-joining (NJ) method [8]

NJ seems to be a method of choice as in obtaining the correct tree, it shows a high performance. When there is an assumption of constant rate of nucleotide substitution then ML method proves to be slightly inferior to NJ, but it is slightly better than other two methods (MP and FM) when among the branches, the evolutionary rate varied drastically [3]. Neighbor joining method (Distance method) reconstructs the phylogenetic tree from evolutionary distance data. It works on the principle that it finds neighbors or pairs of operational taxonomic units (OTUs) and joins them or put into a cluster [8]. Maximum Parsimony is another widely used method for phylogenetic tree reconstruction which is based on sequences [9]. It is character based method.

Alteration in gene/genes or chromosomes is the basal root of any genetic disease. Individuals born as a result of consanguineous union have homozygous segments of their genomes. It is due to inheriting identical ancestral genomic segments through both parents. An increased incidence of recessive diseases within these sibships is one of its consequences [10]. One important example of such type of diseases is Autosomal Recessive Primary Microcephaly (MCPH) which is a rare neurodevelopmental disorder or a neurogenic mitosis disorder. During the process of embryonic neurogensis, generated cerebral cortical neurons are reduced in number. Due to this reason size of MCPH patient’s brain decreases and becomes to 1/3 of its normal volume [11].

Seven loci (MCPH1-MCPH7) with the corresponding genes (MCPH1, WDR62, CDK5RAP2, CEP152, ASPM, CENPJ, and STIL) have been discovered so far from different world populations. It has been proposed that disease phenotype can produced due to mutations in any of genes of MCPH. ASPM and WDR62 gene mutations have a contribution of more than 50% in MCPH Worldwide [11]. WDR62 has been identified as the second most common cause and contributor gene (after ASPM) of MCPH [12].

Computational approaches aims to enhance understanding of biological mechanisms, with primary focus on creating and applying intensive techniques. Phylogenetic analysis and synteny analysis are two most important researches in this discipline. The current study involves this analysis for reported seven Human MCPH genes in order to find out evolutionary relationship and conservation, respectively with respect to various selected ortholog species.

## Materials and methods

**Figure 1:**
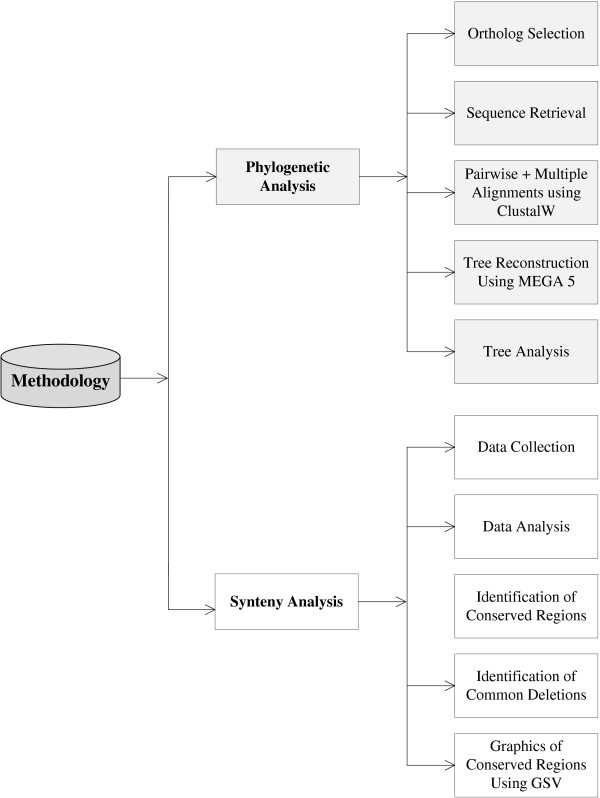
Method and design of the current study.

**Figure 2:**
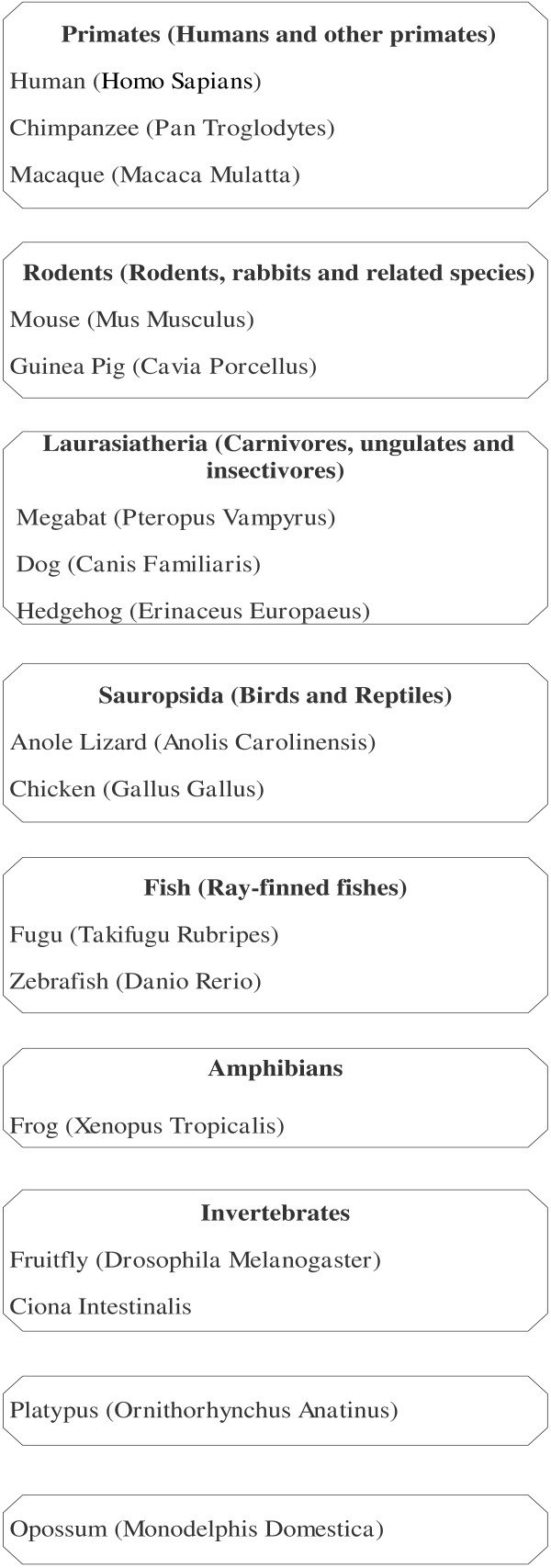
Sixteen ortholog species with respect to human selected for phylogenetic analysis.

Method and design of current study presented by a flow diagram is shown in Figure 1. MEGA5 [13] was used for phylogenetic tree reconstruction. The ClustalW algorithm was used for pair-wise as well as multiple alignment to calculate similarity percentage between sequences and to generate alignment file. Neighbor joining method was used to separately construct trees for all seven genes of MCPH. P-distance was chosen as substitution model. Bootstrap analysis, a computer-based method which assigns accuracy measures to sample estimates [14], was also done to check the reliability of these seven trees. Bootstrap values indicate the confidence and reliability of clusters. Tree topology is tested based on the bootstrap values which further validate the branching pattern. It is an accurate way to control and check stability of results. In the current study bootstrap test uses 1000 replicates and assigns each branch a value ranging from 0 to 100 which gave an idea that how much a sequence is evolutionary closer to each other and also validates each branch. Bootstrap values only greater than 70% were showed. An overall tree for all genes was constructed using maximum parsimony (MP) as it was not constructed through NJ because evolutionary rate was varying drastically among all the genes. NJ shows a high performance in obtaining the correct tree but it’s more sensitive as compare to other methods and do not construct tree when evolutionary rate varies among the genes with high degree. Sixteen ortholog species with reference to Human have been considered in the current study as shown in Figure 2. Sequences of MCPH genes and these orthologs were collected through ensemble database. Sequences of ortholog species were selected after analyzing sequence similarity with Human gene sequence through alignment using BLAST (basic local alignment search tool) [15].

Synteny analysis was performed using Ensemble synteny view in ensemble database [16] and the visual analysis of conserved regions was carried out using web-based genome synteny viewer GSV [17]. For this analysis only four ortholog species of Human have been considered.

## Results

**Figure 3:**
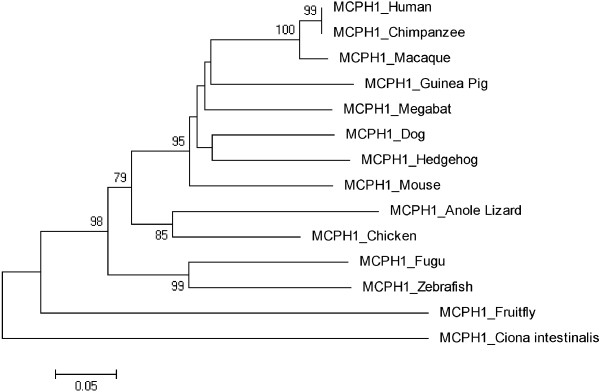
Neighbor Joining (NJ) tree for Human MCPH1 using MEGA5, numbers on branches represent bootstrap values (based on 1000 replications).

**Figure 4:**
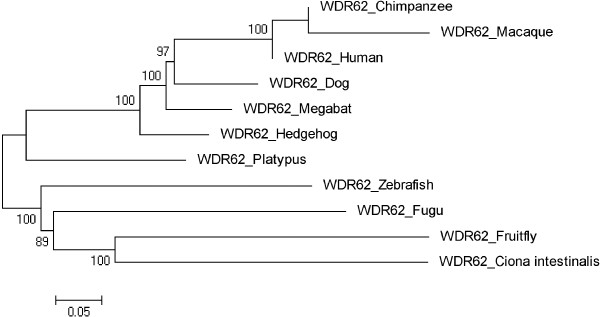
Neighbor Joining (NJ) tree for Human WDR62 using MEGA5, numbers on branches represent bootstrap values (based on 1000 replications).

**Figure 5:**
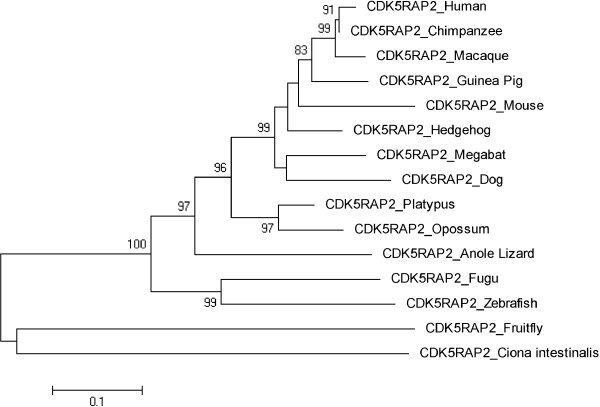
Neighbor Joining (NJ) tree for Human CDK5RAP2 using MEGA5, numbers on branches represent bootstrap values (based on 1000 replications).

**Figure 6:**
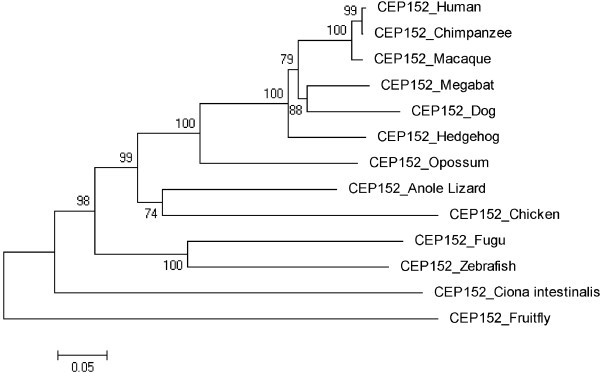
Neighbor Joining (NJ) tree for Human CEP152 using MEGA5, numbers on branches represent bootstrap values (based on 1000 replications).

**Figure 7:**
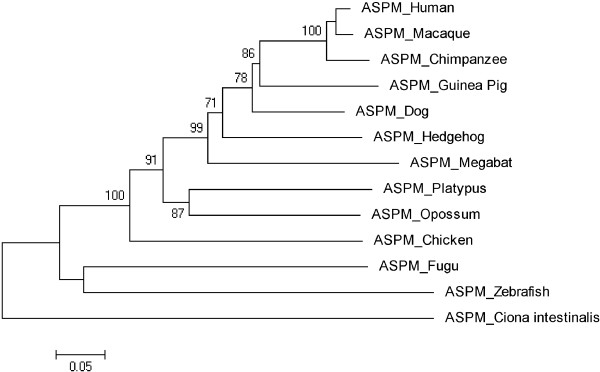
Neighbor Joining (NJ) tree for Human ASPM using MEGA5, numbers on branches represent bootstrap values (based on 1000 replications).

**Figure 8:**
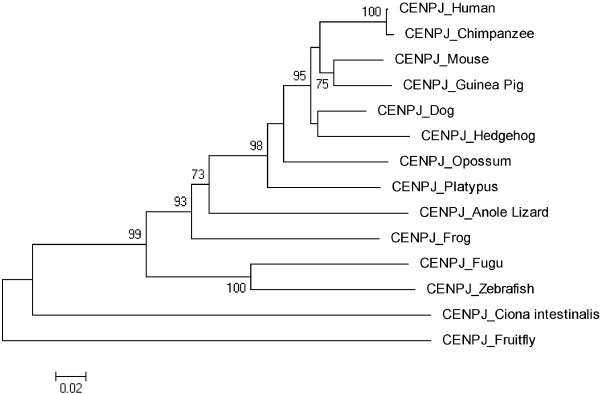
Neighbor Joining (NJ) tree for Human CENPJ using MEGA5, numbers on branches represent bootstrap values (based on 1000 replications).

**Figure 9:**
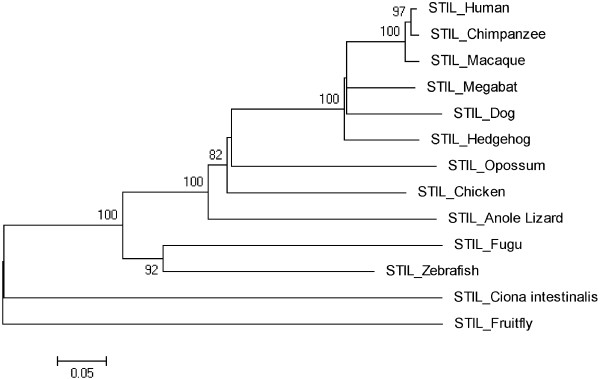
Neighbor Joining (NJ) tree for Human STIL using MEGA5, numbers on branches represent bootstrap values (based on 1000 replications).

Figures 3, 4, 5, 6, 7, 8 and 9 shows trees for seven MCPH genes (MCPH1, WDR62, CDK5RAP, CEP152, ASPM, CENPJ and STIL) constructed through NJ method. These trees show evolutionary relationship among Human and its orthologs selected in the current study. Evolutionary relationship has determined how much species are closely related or deviated from Human. Time of evolution shows the period with which some genetic change occurs and leads to the phenotypical change. It shows the time with which ancestral specie evolve and converts into different species. Evolution could be due to some sort of mutational event, recombination, and selection. Evolutionary time for these constructed trees is; MCPH1 (0.05), WDR62 (0.05), CDK5RAP2 (0.1), CEP152 (0.05), ASPM (0.05), CENPJ (0.02), and STIL (0.05). It shows that CENPJ is evolving rapidly as compare to others. Maximum evolutionary rate is of gene CDK5RAP2 as compare to other genes i.e. 0.1 which provides us the hypothesis that it is evolving with least rate as compare to others. All these trees are reconciling the species divergence time. The description for each tree is given below:

### MCPH1

Neighbor joining tree for MCPH1 is shown in Figure 3. This is reconstructed tree after deleting three sequences (Opossum, Frog and Platypus) from original tree. These sequences were not according to the time of divergence hence removed from the tree. The reconstructed tree (Figure 3) is reconciling the species divergence time except mouse which has shown instant divergence from Human. According to tree, Human and Chimpanzee are in one cluster with a bootstrap value of 99 while Macaque is close to Human/Chimpanzee with 100 as a bootstrap value. Vertebrates Ciona intestinalis and Fruitfly are as outgroup in this tree. Evolutionary time for the tree is 0.05.

### WDR62

The tree was initially constructed using fourteen ortholog species of Human. Two orthologs Anole Lizard and Opposum have diverged sequences as compare to the rest of species due to which these have been excluded. Chicken, Frog, Guinea Pig and Mouse are not according to the time of divergence. These four orthologs were deleted and the tree was reconstructed. The reconstructed tree is shown in Figure 4 and it is reconciling the species divergence time. According to this tree Human is closely related to Macaque and Chimpanzee cluster with the bootstrap value of 100. Invertebrates Ciona intestinalis and Fruitfly are in one cluster with 100 as a bootstrap value. 0.05 is the evolutionary value of tree.

### CDK5RAP2

Neighbor joining tree for CDK5RAP2 is shown in Figure 5. Frog and Chicken were removed from the initial tree as they were not according to the time of divergence. The tree was reconstructed after deleting these two orthologs. The reconstructed tree (Figure 5) having same results for Human and Chimpanzee cluster with bootstrap value of 91. Evolutionary time for tree is 0.1. According to tree invertebrates Ciona intestinalis and Fruitfly are in one cluster and are as out group. Zebrafish and Fugu are in one cluster with a bootstrap value of 99. Similarly Opossum/Platypus are making cluster with 97 as bootstrap value.

### CEP152

In the initial tree, Frog, Platypus, Mouse and Guinea Pig were not according to the time of divergence hence they were deleted from the tree. The tree was reconstructed after deleting these sequences and is shown in the Figure 6. The reconstructed tree is reconciling the species divergence time. According to this tree, invertebrates Ciona intestinalis and Fruitfly are as out group. Human and Chimpanzee are in one cluster (have same ancestor) with 99 as a bootstrap value indicating reliability of this cluster. Macaque and ancestor of Human/Chimpanzee are evolving from the same ancestor. Macaque is evolving with a bootstrap value of 100 and is closely related to the cluster of Human/Chimpanzee. Zebrafish and Fugu are in one cluster with a bootstrap value of 100. Similarly Chicken/Anole Lizard and Dog/Megabat are making clusters with 74 and 88 as bootstrap values, respectively. Evolutionary time is 0.05 for the tree.

### ASPM

Anole Lizard, Fruitfly, Frog and Mouse have been deleted from the tree constructed initially as they were not according to the time of divergence. The tree was reconstructed after deleting these four orthologs and is shown in Figure 7 having rate of evolution as 0.05. In this tree Human is making cluster with Macaque instead Chimpanzee. Chimpanzee is evolving with a bootstrap value of 100 and is close to Human/Macaque cluster. Opossum and Platypus are making cluster with 87 as bootstrap value.

### CENPJ

Two orthologs Chicken and Macaque have diverged sequences as compare to the rest of species due to which these have been excluded. Megabat is deleted as it is not according to the time of divergence. After deletion tree was reconstructed as shown in Figure 8 and has evolutionary time of 0.02. According to this tree, invertebrates Ciona intestinalis and Fruitfly are as outgroup. Human is making cluster with Chimpanzee with 100 as a bootstrap value indicating the reliability of cluster. Human/Chimpanzee cluster in original tree is evolving from the same ancestor with a bootstrap value of 99 while in reconstructed tree their bootstrap value is 100. Zebrafish/Fugu and Guinea Pig/Mouse are making cluster with bootstrap values 100 and 75, respectively.

### STIL

From the initial tree, Frog, Platypus, Guinea Pig and Mouse were deleted and tree was reconstructed after deleting these four orthologs and it is shown in Figure 9. The rate of evolution is 0.05. According to this tree invertebrates Ciona intestinalis and Fruitfly are in one cluster and are as out group. Zebrafish and Fugu are making a cluster with a bootstrap value of 92. In this tree Human/Chimpanzee is in one cluster with 97 as a bootstrap value.

### Combined tree for seven MCPH genes

An overall tree for all the seven genes constructed through MP method is shown in Additional file 1. This tree shows that ASPM Ciona intestinalis and CEP152 Ciona intestinalis are in one cluster, which shows that there are two copies of same gene. Hence one copy i.e. ASPM Ciona intestinalis has been deleted. All sequences of gene STIL have been deleted as these sequences seem too divergent. The sequences; MCPH1 Opposum, MCPH1 fruitfly, CEP152 fruitfly, CENPJ Megabat, CENPJ Opposum, CENPJ Platypus, CENPJ chicken, CDK5RAP2 Fruitfly, and CDK5RAP2 Ciona intestinalis were not according to the time of divergence, hence these have also been deleted from the tree.

### Tree reconstruction

We have reconstructed tree by removing all of the sequences mentioned above. The tree which is reconciling the species divergence time is given in Additional file 2.

### Tree analysis

**Figure 10:**
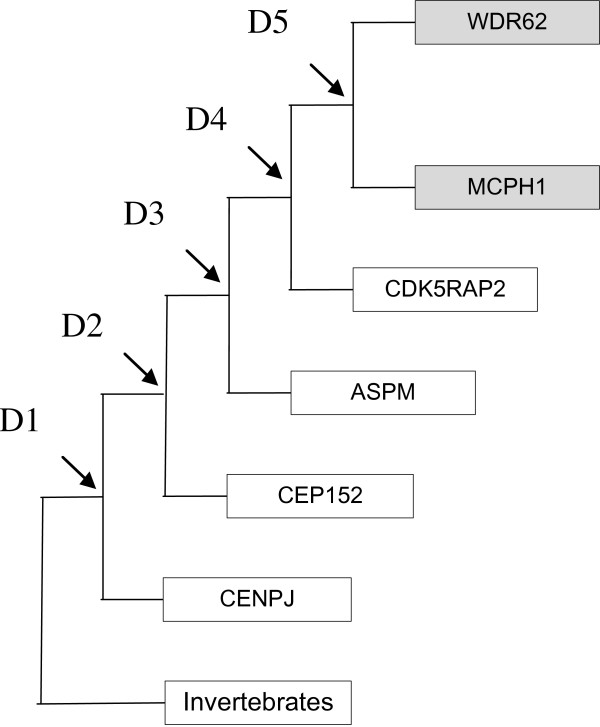
Duplication events (D1→D5; indicated by arrows) in maximum parsimony (MP) tree for seven human MCPH genes.

According to our understanding, there are five duplication events as are shown in tree (Figure 9). Four duplications takes place before vertebrates and invertebrates divergence and only one duplication takes place after vertebrate and invertebrate divergence. First duplication occurs after vertebrate and invertebrate divergence (Invertebrate CENPJ_Ciona intestinalis evolve first and then first duplication takes place). Hypothetical ancestor had a gene (MCPH1/ WDR62/ CDK5RAP2/ CEP152/ ASPM/ CENPJ), on first duplication it became (CENPJ) and (ancestor of genes MCPH1/ WDR62/ CDK5RAP2/ CEP152/ ASPM). All the remaining duplications occur before vertebrate and invertebrate divergence. As a result of second duplication in the gene produced as a result of first duplication (i.e. CENPJ), CEP152 and ancestor of (MCPH1/ WDR62/ CDK5RAP2/ ASPM) formed. Third duplication which occurs in the ancestral gene of (MCPH1/ WDR62/ CDK5RAP2/ ASPM) forms ASPM and (ancestor of MCPH1/ WDR62/ CDK5RAP2). Fourth duplication occurs in the ancestral gene of MCPH1/ WDR62/ CDK5RAP2 which forms two copies, one is CDK5RAP2 and second ancestral gene of MCPH1/ WDR62. Finally fifth duplication occurs in ancestral gene of MCPH1/ WDR62 which forms two copies i.e. MCPH1 and WDR62. This shows that MCPH1 and WDR62 are closely related to each other (Highlighted in Figure 10) as these genes are in one cluster.

### Genome synteny analysis

In order to find out the genomic elements that are functionally conserved, we find out set of genomic features (genes or loci) that are conserved, in the same relative ordering on a set of homologous chromosomes (of human and its four orthologs). We studied conservation of human 15 genes (both upstream and downstream of seven MCPH genes) with genes of its four orthologs. Data collected from ensembl syntenyview in ensembl database and its summary is given in Additional file 3.

**Figure 11:**
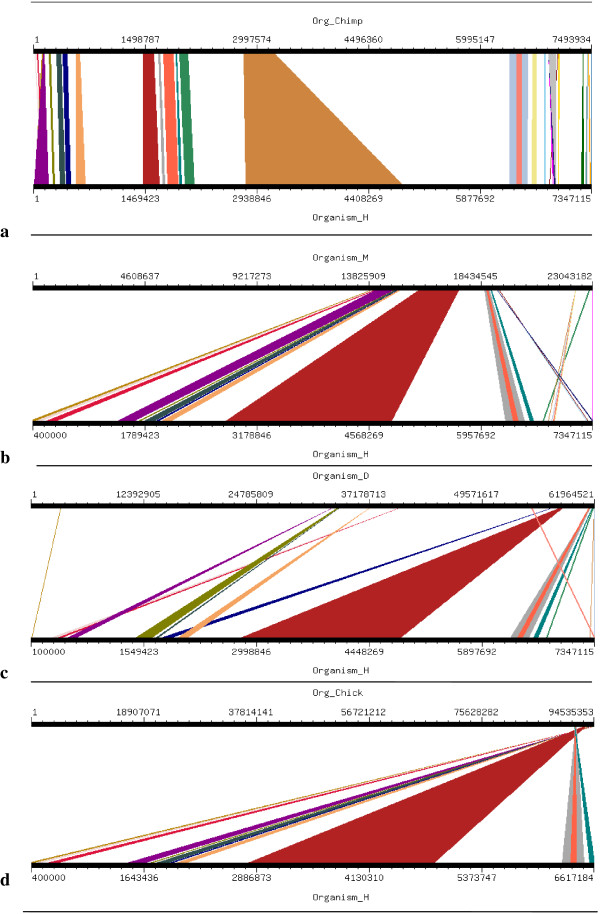
Results of GSV for Human MCPH1 showing conserved regions; a) Org_Chimp (Chimpanzee) vs Organism_H (Human); b) Organism_M (Mouse) vs Organism_H (Human); c) Organism_D (dog) vs Organism_H (Human); d) Org_Chick (Chicken) vs Organism_H (Human).

**Figure 12:**
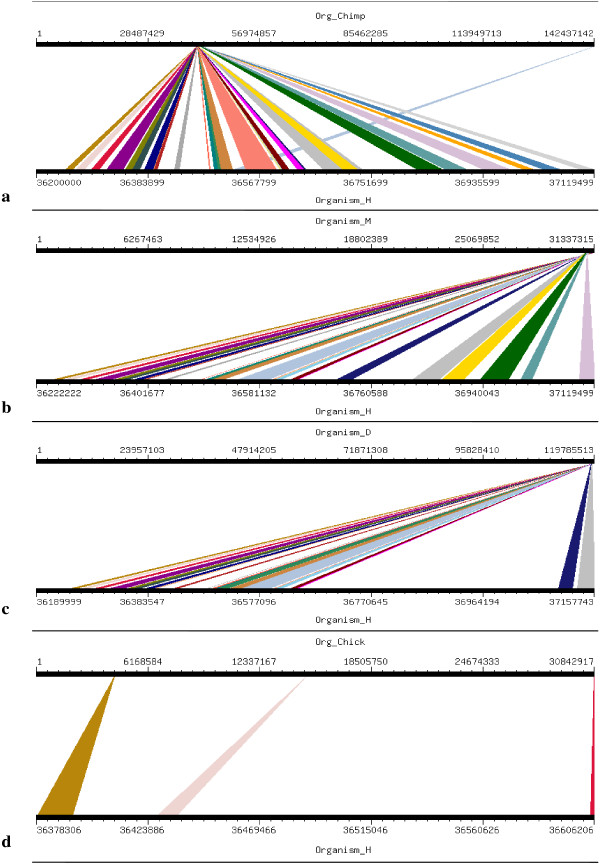
Results of GSV for Human WDR62 showing conserved regions; a) Org_Chimp (Chimpanzee) vs Organism_H (Human); b) Organism_M (Mouse) vs Organism_H (Human); c) Organism_D (dog) vs Organism_H (Human); d) Org_Chick (Chicken) vs Organism_H (Human).

**Figure 13:**
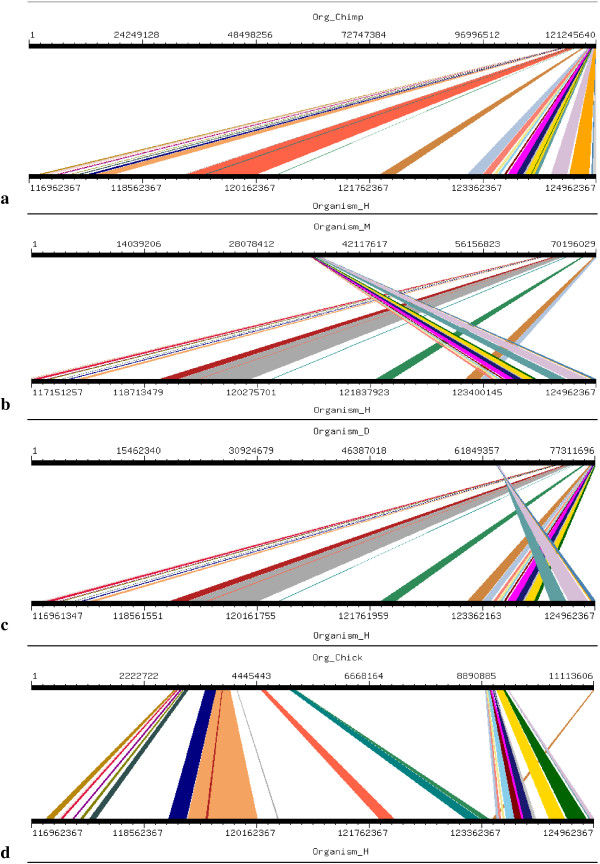
Results of GSV for Human CDK5RAP2 showing conserved regions; a) Org_Chimp (Chimpanzee) vs Organism_H (Human); b) Organism_M (Mouse) vs Organism_H (Human); c) Organism_D (dog) vs Organism_H (Human); d) Org_Chick (Chicken) vs Organism_H (Human).

**Figure 14:**
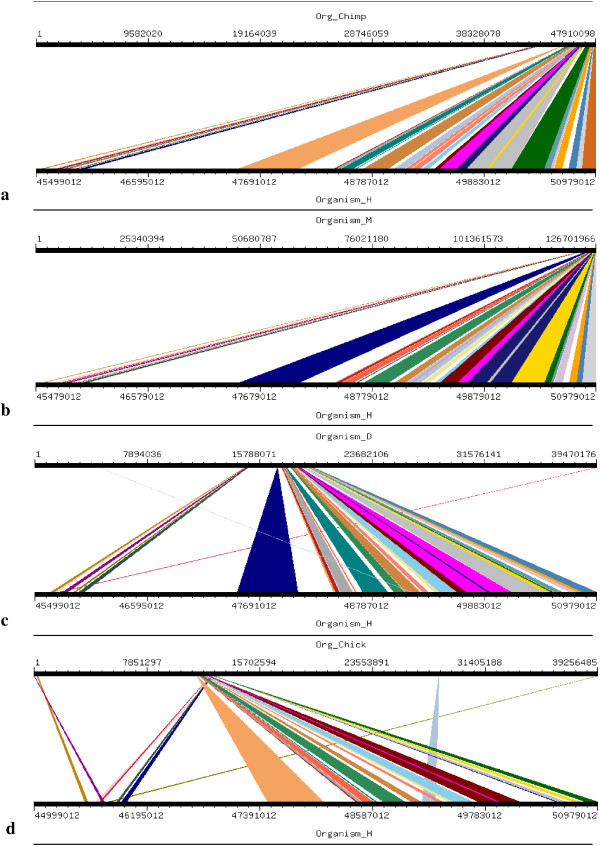
Results of GSV for Human CEP152 showing conserved regions; a) Org_Chimp (Chimpanzee) vs Organism_H (Human); b) Organism_M (Mouse) vs Organism_H (Human); c) Organism_D (dog) vs Organism_H (Human); d) Org_Chick (Chicken) vs Organism_H (Human).

**Figure 15:**
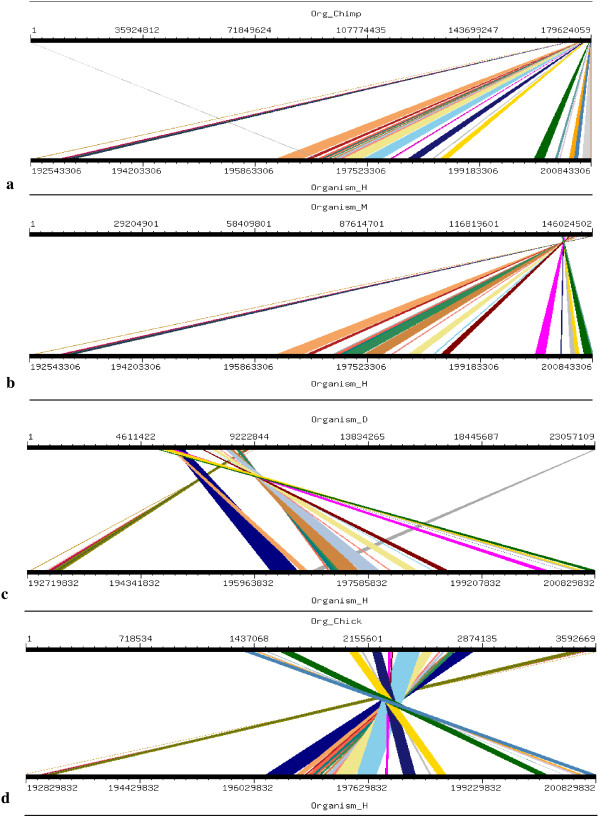
Results of GSV for Human ASPM showing conserved regions; a) Org_Chimp (Chimpanzee) vs Organism_H (Human); b) Organism_M (Mouse) vs Organism_H (Human); c) Organism_D (dog) vs Organism_H (Human); d) Org_Chick (Chicken) vs Organism_H (Human).

**Figure 16:**
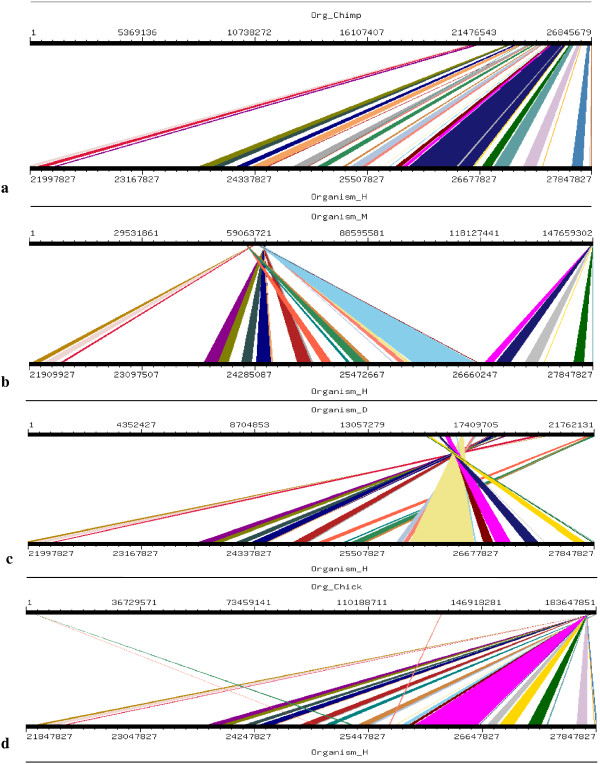
Results of GSV for Human CENPJ showing conserved regions; a) Org_Chimp (Chimpanzee) vs Organism_H (Human); b) Organism_M (Mouse) vs Organism_H (Human); c) Organism_D (dog) vs Organism_H (Human); d) Org_Chick (Chicken) vs Organism_H (Human).

**Figure 17:**
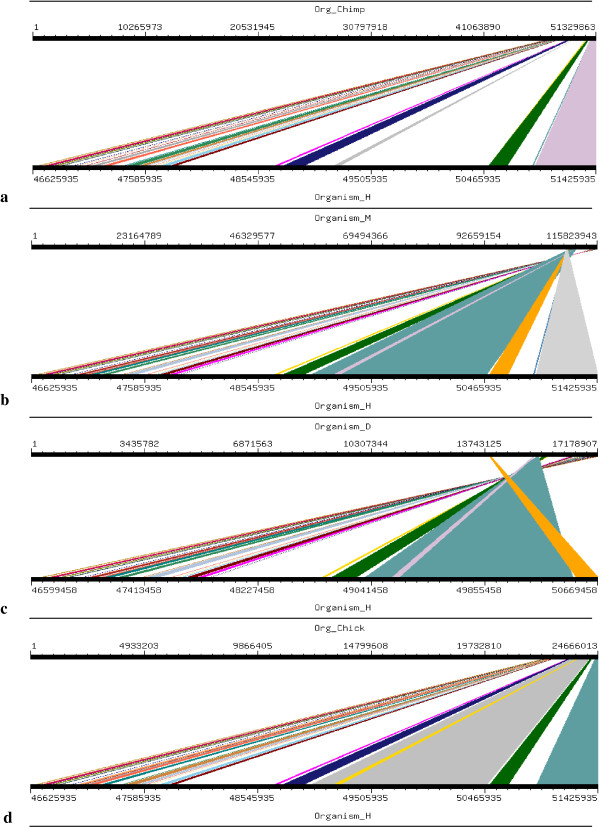
**Results of GSV for Human STIL showing conserved regions; a) Org_Chimp (Chimpanzee) vs Organism_H (Human); b) Organism_M (Mouse) vs Organism_H (Human); c) Organism_D (dog) vs Organism_H (Human); d) Org_Chick (Chicken) vs Organism_H (Human).**

Four orthologs which have been considered for this study are Chimpanzee (Pan troglodytes), Mouse (Mus musculus), Dog (Canis familiaris) and Chicken (Gallus gallus). Mouse, Dog and Chicken were selected as they have a sequence coverage of at least 7-folds. Second reason for which these four orthologs were selected was that we tried to include those closely related as well as those divereged with respect to Human. By this we were able to clearly demonstrate the presence and absence of conserved synteny between Human and its orthologs. Conserverd regions were also generated using genome synteny viewer GSV web server which produced graphical representations and facilitated the quick visualization of conserved regions in the form of colored blocks with the ruler indicating positions of these conserverd regions (Figures 11, 12, 13, 14, 15, 16 and 17). In all genes majority of the portion is conserved between orthologs and human as indicated by colored blocks in relevance to human. Our analysis showed that in WDR62 majority of the portion is conserved among two orthologs (Chimpanzee and Mouse) in relevance to human then with some deletions in Dog and very poor conservation found with Chicken with only three conserved regions. Changes which lead towards the evolution of these organisms are given in Additional file 4. Synteny analysis showed that in MCPH1 there exist only three deletions in Chimpanzee while maximum deletions (i.e. 18) exist in Chicken in relevance to Human according to our synteny location map (data from ensembl syntenyview in ensembl database). This indicates Human and Chimpanzee are closely related. In WDR62 there are two deletions in Chimpanzee and five in mouse. Maximum deletions (i.e. 28) exist in case of chicken ortholog with respect to Human. Similarly, according to our synteny location map, in CDK5RAP2, genes are conserved in all four orthologs in relevance to Human as there are few deletions (i.e. 2, 3, 3, and 5 in Chimpanzee, Mouse, Dog and Chicken, respectively). In CEP152, Human is closely related to Chimpanzee with only one deletion. While Mouse, Dog and Chicken are also conserved with only few deletions in genes i.e. 2, 3, and 7, respectively. Human is more conserved with Chimpanzee in relevance to ASPM gene with only one deletion of gene i.e. CFHR3. Chicken is also closely related to Human after Chimpanzee with six deletions. Highly conserved synteny has been observed for Human and Chimpanzee in case of CENPJ with no deletion. Chicken and Human are also closely related with only two deletions while there are five deletions in Mouse and Dog in relevance to Human. Human and Mouse are conserved in case of gene STIL having only two deletions while there are 4, 5 and 6 deletions in orthologs Dog, Chimpanzee and Chicken, respectively. This has been observed according to our synteny location maps (data obtained through ensembl syntenyview in ensembl database).

According to Additional file 4, common deletions in four orthologs in relevance to Human are two in case of MCPH1 i.e. DEFA6, SPAG11B. In case of CEP152, only one common deletion in four orthologs occurs i.e. RP11-90J19.1 while in remaining five genes no common deletions have been found. All seven MCPH genes (MCPH1, WDR62, CDK5RAP2, CEP152, ASPM, CENPJ, and STIL) are present in four ortholog species (Chimpanzee, Mouse, Dog and Chicken) in relevance to Human except WDR62 which is deleted in Chicken only. This shows the importance of MCPH genes in these species.

## Discussion

An important research area in the field of computational biology is phylogenetic analysis which aims to study and estimate evolutionary relationship between organisms. Evolution is the change that leads towards the diversity. This diversity can be at any biological level including species, organisms, and also at molecular level i.e. DNA and Proteins.

Trees for seven MCPH genes (MCPH1, WDR62, CDK5RAP, CEP152, ASPM, CENPJ and STIL) were constructed through NJ method which showed evolutionary relationship among Human and its orthologs. Through this evolutionary relationship it has been determined how much species are closely related or deviated from Human. Rate of evolution for constructed trees showed that CENPJ (0.02) evolving rapidly as compare to rest of the genes. CDK5RAP2 with maximum evolutionary rate (i.e. 0.1) showed that this gene evolving with least rate as compare to others MCPH gene. All MCPH trees are reconciling the species divergence time. Bootstrap values in all trees have helped in the validation of clusters in the tree. These values clearly indicate the reliability of clusters. In WDR62, Human is closely related to the cluster of Macaque and Chimpanzee (with bootstrap value of 100). Similarly Human is making cluster with Chimpanzee in MCPH1, CDK5RAP2, CEP152, CENPJ and STIL with bootstrap values of 99, 91, 99, 100, and 97 respectively, while it is making cluster with Macaque in ASPM with 50 as a bootstrap value. Chimpanzee is evolving with a bootstrap value of 100 in ASPM tree and is close to Human/Macaque cluster. Function of MCPH genes present in Human ortholog species is same as function of Human MCPH genes and this function in these species remains intact unless and until mutation comes. Only the difference is in sequences of their genes (which lead to phenotypic changes as well) and through our results we demonstrated the level of difference. Our results showed how close an ortholog speice is to the query (Human) in reference to each MCPH gene. In case of every MCPH gene, ortholog species present in cluster with Human or near the cluster of Human were most likely (with less difference in their sequences) as compare to those which were present away from Human in the tree. In the combined tree of MCPH genes, five duplications have been observed dividing ancestral gene into descendent genes. Two genes MCPH1 and WDR62 found to be closely related evolved at the end as a result of fifth duplication and are in one cluster. Four duplications have been observed before vertebrates and invertebrates divergence and only one duplication took place after vertebrate and invertebrate divergence i.e. first duplication.

Syntenic relationship for all MCPH genes indicated that maximum conservation of Human has been found with Chimpanzee in five genes: MCPH1, WDR62, CDK5RAP2, CEP152, ASPM, and CENPJ while with Mouse in case of gene STIL. Highly conserved synteny has been observed for Human and Chimpanzee in case of CENPJ with no deletion.

## Conclusion

Current study shows that CENPJ is evolving rapidly as compare to others. Maximum evolutionary rate is of gene CDK5RAP2 provides us the hypothesis that it is evolving with least rate as compare to others. In WDR62, Human is closely related to the cluster of Macaque and Chimpanzee. Similarly Human is making cluster with Chimpanzee in MCPH1, CDK5RAP2, CEP152, CENPJ and STIL, while it is making cluster with Macaque in ASPM. The closest specie of Human in our analysis have been found to be Chimpanzee as maximum genes are showing their cluster and hence direct relationship of both ortholog species. According to our understanding, there are five duplication events in tree. Four duplications takes place before divergence of vertebrates and invertebrates and only one duplication is taking place after vertebrate and invertebrate divergence. Duplication events showed that MCPH1 and WDR62 are closely related to each other and evolved at the end as compare to other genes. According to synteny analysis maximum conservation of Human has been found with Chimpanzee in MCPH1, WDR62, CDK5RAP2, CEP152, ASPM, and CENPJ and with Mouse in case of gene STIL.

From our present results, we hypothesized that due to having closest relationship, it is possible that mutations can affect Chimpanzee (closest Human relative according to our results) likewise as these affect Human and can lead to microcephaly. It also shows genes of microcephaly in closest relative species (Human/Chimpanzee) have maximum similarity in their sequences and share a close syntenic relationship. Conservation shows that apart from sequence similarity, function of MCPH genes in closely related species is also same and this function disrupts as a result of mutation and hence leads to the diseased state.

## Competing interests

Being authors we declare that we have no competing interests.

## Authors’ contributions

The work presented here was carried out in collaboration between both authors. AM defined the research theme, designed methods, analyzed the data, interpreted the results and helped in writing the paper. SR carried out all the work, analyzed results and wrote the paper. Both authors have contributed to, seen, read and approved the manuscript.

## Supplementary Material

Additional file 1 Maximum Parsimony (MP) Tree for Seven Human MCPH Genes using MEGA5. Click here for file

Additional file 2 Reconstructed Maximum Parsimony (MP) Tree for Seven Human MCPH Genes using MEGA5. Click here for file

Additional file 3 Summary of Data Collected for Synteny Analysis. Click here for file

Additional file 4 Common Deletions in Four Orthologs in Reference to Human. Click here for file
