# Supplementary figures and images for: Exploring evolution of brain genes involved in microcephaly through phylogeny and synteny analysis
Source: Theor Biol Med Model. 2013 Oct 22;10:61. doi: 10.1186/1742-4682-10-61 (PMC4015606; doi:10.1186/1742-4682-10-61)

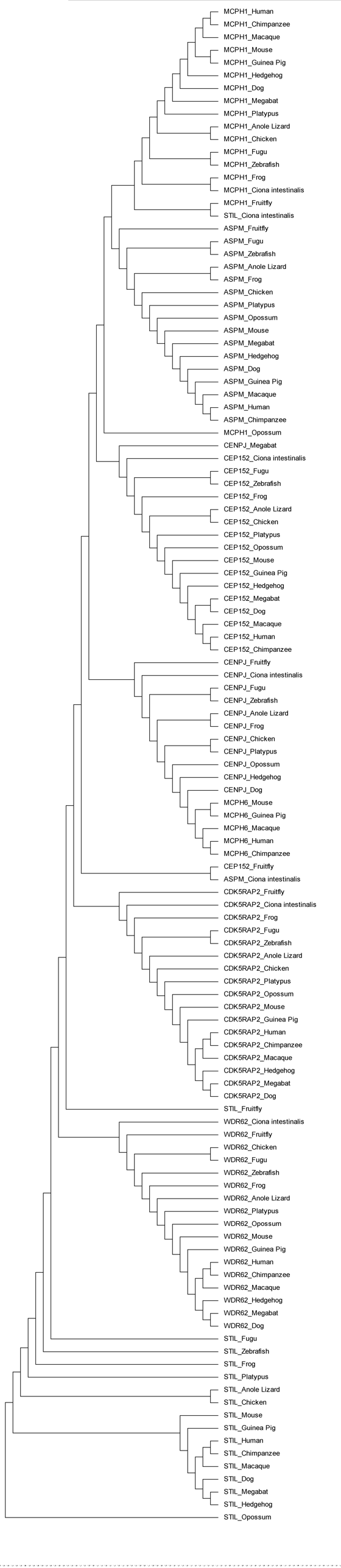


**Maximum Parsimony (MP) Tree for Seven Human MCPH Genes using MEGA5**

Supplement: Additional file 1 — Maximum Parsimony (MP) Tree for Seven Human MCPH Genes using MEGA5. [file 1742-4682-10-61-S1.doc]

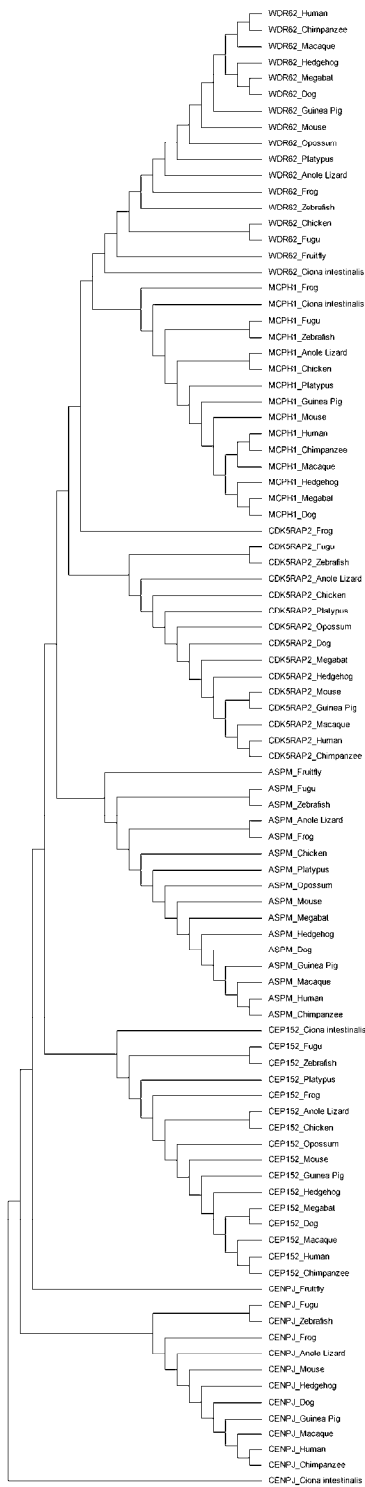

**Reconstructed Maximum Parsimony (MP) Tree for Seven Human MCPH Genes using  
MEGA5**

Supplement: Additional file 2 — Reconstructed Maximum Parsimony (MP) Tree for Seven Human MCPH Genes using MEGA5. [file 1742-4682-10-61-S2.pdf]
